# Inflammatory response and MAPK and NF-κB pathway activation induced by natural street rabies virus infection in the brain tissues of dogs and humans

**DOI:** 10.1186/s12985-020-01429-4

**Published:** 2020-10-20

**Authors:** Shu Qing Liu, Yuan Xie, Xin Gao, Qian Wang, Wu Yang Zhu

**Affiliations:** 1 Key Laboratory of Medical Virology, Ministry of Health, National Institute for Viral Disease Control and Prevention, NHC Key Laboratory of Biosafety, Chinese Center for Disease Control and Prevention, No.155 Changbai Road, Changping District, Beijing, 102206 People’s Republic of China; 2 Pathogenic Microbiology Institute, Tianjin Centers for Disease Control and Prevention, Tianjin, 300011 People’s Republic of China; 3 College of Global Change and Earth System Science, Beijing Normal University, Beijing, 100875 People’s Republic of China

**Keywords:** Street rabies virus, Inflammatory response, Mitogen-activated protein kinase, NF-kappaB pathway, Natural infection

## Abstract

**Background:**

Street rabies virus (RABV) usually infects hosts at peripheral sites and migrates from motor or sensory nerves to the central nervous system. Several studies have found that inflammation is mild in a mouse model of street RABV infection. However, the pathogenetic mechanisms of street RABV in naturally infected dogs or humans are not well understood.

**Methods:**

Brain tissues collected from 3 dogs and 3 humans were used; these tissue samples were collected under the natural condition of rabies-induced death. The inflammatory response and pathway activation in the brain tissue samples of dogs and humans were evaluated by HE, IHC, ARY006, WB and ELISA. The clinical isolate street RABV strains CGS-17 and CXZ-15 from 30 six-week-old ICR mice were used to construct the mouse infection model presented here.

**Results:**

Neuronal degeneration and increased lymphocyte infiltration in the cerebral cortex, especially marked activation of microglia, formation of glial nodules, and neuronophagy, were observed in the dogs and humans infected with the street RABV strains. The various levels of proinflammatory chemokines, particularly CXCL1, CXCL12, CCL2, and CCL5, were increased significantly in the context of infection with street RABV strains in dogs and humans in relation to healthy controls, and the levels of MAPK and NF-κB phosphorylation were also increased in dogs and humans with natural infection. We also found that the degrees of pathological change, inflammatory response, MAPK and NF-κB signaling pathway activation were obviously increased during natural infection in dogs and humans compared with artificial model infection in mice.

**Conclusion:**

The data obtained here provide direct evidence for the RABV-induced activation of the inflammatory response in a dog infection model, which is a relatively accurate reflection of the pathogenic mechanism of human street RABV infection. These observations provide insight into the precise roles of underlying mechanisms in fatal natural RABV infection.

## Background

Rabies is a fatal zoonotic disease caused by the rabies virus (RABV), and infection leads to encephalomyelitis in mammalian species [1]. Street RABV usually infects hosts at peripheral sites and migrates from motor or sensory nerves to the central nervous system (CNS), and it employs a series of strategies to evade host immune responses [2]. Several studies have found that inflammation is mild in a mouse model of street RABV infection, which seems to fail to trigger sufficient proinflammatory chemokine and cytokine production, resulting in relatively little cell infiltration and neuronal inflammation in the CNS [3].

Cytokines and chemokines orchestrate a multicellular immune response though microglial, monocyte, and astrocyte activation in response to viral infection, which subsequently recruits peripheral leukocytes into the CNS [4]. Fu et al. showed that the induction of chemokines/cytokines and their roles in orchestrating downstream immune events are a key feature in clearing RABV from the CNS [5]. The ability of RABV to evade the expression of chemokines/cytokines contributes to virulence and pathogenicity [3]. Most street RABV strains evade the host innate immune system, and neuronal pathology or damage to the CNS is limited in street rabies patients with only mild inflammation [6]. Suja MS et al. also reported that despite dramatic clinical symptoms and outcomes, surprisingly little tissue damage or neuronal pathology has been observed in the brains of rabies patients [7]. However, in some murine or dog experimental models infected with street RABV strains, T cell and mononuclear cell infiltration in the CNS has been observed together with severe encephalitis in the terminal stage of infection [8]. Increasing evidence suggests that excessive immune responses may be associated with pathological processes and that some chemokines (such as CXCL10 and CCL5) are associated with excessive inflammation in the CNS, contributing to the increased pathogenicity observed in neurological diseases in mice infected with attenuated RABV [9].

The mitogen activated protein kinase (MAPK) pathway is a major cellular signaling pathway that converts extracellular signals into a multitude of cellular responses and regulates gene expression, the immune response and so on; this pathway is activated by a variety of chemical and physical stimuli and diverse groups of viruses. MAPK cascades include the p38 kinase, c-Jun-N-terminal kinase (JNK) and extracellular-regulated kinase (ERK)1/2 pathways [10]. p38 activation leads to the phosphorylation of a large number of transcription factors that regulate proinflammatory mediators, such as IL-8, TNF-α, RANTES, and CXCL10, following viral infection. Activation of JNK is important for apoptosis, and transient activation also contributes to the survival of cells and replication of viruses, such as HSV-1, DENV, IAV and VZV. ERK1/2 molecules serve as a reasonable target for the modulation of viral multiplication [11]. MAPKs are involved in many facets of cellular regulation and immune responses and possibly contribute to cytokine production by interfering with the nuclear factor kappa B (NF-κB) signaling pathway [10]. MAPK phosphorylates a large number of substrates and induces the activation of transcription factors such as NF-κB p65, c-Jun and STAT1, which play pivotal roles in regulating the immune response to viral infection and cytokines, and the expression of most chemokines is regulated primarily at the level of transcription through the activation of NF-κB and interferon (IFN) regulatory factors [11].

Laboratory-adapted RABV infection triggers the activation of signaling pathways mediated by MAPK and NF-κB in microglia, and the induction of the iNOS and CXCL10 genes in RABV-stimulated macrophages is mediated through the activation of ERK1/2 [12]. There are known biological differences between street and attenuated strains of RABV that can influence the host’s ability to initiate apoptosis in infected cells and mount innate immune responses, but in most cases, the molecular basis for these differences is not clear [13]. Little tissue damage or histopathological evidence of nervous system dysfunction has been observed in animals dying of rabies [14]. Despite years of research, the understanding of the pathogenicity of rabies virus infection has mostly come from infection of experimental animals with laboratory-adapted RABV strains, but the pathological changes observed in these experimental infection models greatly differ among various virus strains [15], and the abnormalities caused by laboratory-adapted RABV strain infection are not present in all infected animals, which particularly indicates that these models do not reflect the pathogenetic process and mechanism of animals infected with street rabies virus under natural conditions [14]. Therefore, we detected the histopathological changes, inflammatory response and signaling pathway mechanism in dogs and humans upon infection with two street rabies virus strains under natural conditions.

## Materials and methods

### Ethics statement

The procedure for the collection of human brain specimens was approved by the Ethical Committee of the National Institute of Viral Disease Control and Prevention, Chinese Center for Disease Control (CDC), which is the national referral center for rabies diagnosis (permission number: IVDC2018-020). All of the animal experiments were carried out in accordance with the recommendations in the Guide for the Care and Use of Laboratory Animals of the Ministry of Science and Technology of China and approved by the Institutional Animal Care and Use Committee, Chinese CDC (Permission Number: 20180222004).

### Viral strains and animal studies

The street RABV strains CGS-17 and CXZ-15 were originally isolated from the brain tissues of a dog from Gan Su province in 2016 and a human with rabies in Tibet [16]. Brain tissues collected from 3 dogs and 3 humans were used. Briefly, the clinical human strain was from a person in Tibet who died in the local hospital; he was bitten on his left wrist by a stray dog approximately two months earlier, did not seek vaccination after the event, and exhibited clinical symptoms consistent with rabies 6 days prior to death. This patient was reported as a suspected rabies case to the NDRS of the Chinese CDC. The family agreed to collection of his brain tissue, which was sent to the Chinese CDC rabies laboratory (our laboratory) for laboratory diagnosis and subsequently confirmed as rabies positive. The viral variants in cases of human rabies appear to be very similar and are placed within the China IV lineage, as described in our previous study [16]. The clinical dog cases were chosen from human cases associated with exposure to aggressive dogs; brain tissues were collected from these dogs upon syndrome manifestations, and the dogs were confirmed to be rabid in a laboratory for viral testing following standardized procedures. There were three dog and three human brain samples. Seven-day-old ICR suckling mice and 6-week-old ICR mice were purchased from the Institute of Laboratory Animal Medicine at the Chinese Academy of Medical Sciences (CAMS & PUMC, Beijing, China). The mice were randomly divided into three groups (10 mice per group). All of the procedures were conducted in accordance with the Guidelines for the Medical Laboratory Animal (1998) from the Ministry of Health, China. A 10% (w/v) suspension was prepared by homogenizing the brain tissues of dogs or humans in Dulbecco's modified Eagle medium (Invitrogen, Grand Island, NY, USA). The homogenate was centrifuged at 1500 g for 10 min (at 4 °C) to remove debris, and the supernatant was collected and stored at −80 °C. Seven-day-old ICR suckling mice were inoculated with 10 μL of viral inoculum via the intracerebral (i.c.) route. When moribund, the mice were euthanized, and the brains were removed under sterile conditions. The titer of the 10% (w/v) viral supernatant was determined by inoculating the viral inoculum of each strain into 7-day-old ICR suckling mice via the i.c. route, and the 50% mouse i.c. lethal dose (MICLD50) was calculated using the Reed-Muench method. Viral stocks were prepared as previously described [17]. Six-week-old mice (n ≥ 3 per group) were intramuscularly infected with RABV strains (CGS-17 and CXZ-15; 5.6 × 10^3^ FFU; 25 μl in PBS) in the gastrocnemius muscle of the right thigh [14]. The same volume of sterile PBS was used for mock controls. Brains were removed separately and stored according to the requirements of the test.

### Histopathology and Immunohistochemistry

The dog and human brain tissues showing cortex death in street RABV strain CGS-17 and CXZ-15 infection were removed and fixed in 10% formaldehyde/PBS (v/v) fixation buffer for hematoxylin and eosin (HE) and immunohistochemistry (IHC) assays [18]. Due to the scarcity of healthy human brain tissue, uninfected dog brain tissues served as the control group. ICR mice were challenged as described above with CGS-17 and CXZ-15 and evaluated by HE staining [19]. In the IHC assay, fixed brains infected with CGS-17 and CXZ-15 as described above were prepared as previously described. The following antibodies were used: anti-p-p38 (#4631; Cell Signaling Technology), anti-p-ERK1/2 (#4370; Cell Signaling Technology), anti-p-JNK (#4668; Cell Signaling Technology), and anti-p-NF-κB (#3033; Cell Signaling Technology). Quantitative analysis of immunopositive cells was performed by counting both positive and negative cells (minimum of 1000 cells) in 15–20 representative high-power fields [19]. Both the histopathology and immunohistochemistry slides were read and interpreted by the same pathologist.

### Inflammatory antibody array and ELISA

The Proteome Profiler™ Array Mouse Cytokine Array Panel A (ARY006, R&D Systems, Minneapolis, MN, USA) was used to measure the protein expression levels of 40 cytokines and chemokines in the brain tissues. The cytokines and chemokines are provided in Additional file 1: Figure S1. Quantitation of protein concentrations was carried out using a total protein assay. The array was inoculated with 300 μg proteins, and samples were treated according to the product specification. Briefly, protein samples were diluted and mixed with a cocktail of biotinylated detection antibodies and then incubated with a mouse chemokine or cytokine array membrane. After washing, streptavidin-conjugated horseradish peroxidase and chemiluminescent detection reagents were added. Array images showing chemiluminescence signals were obtained using an LAS-4000 imaging system (Fujifilm, Tokyo, Japan) and analyzed by densitometry for integral optical density using ImageJ software. The optical density of each pair of chemokine or cytokine spots was normalized to the corresponding positive control spots [20].

ELISA kits (R&D Systems) were used to measure CXCL1, CXCL12, CCL2, and CCL5 levels in brain tissues of mice infected with CGS-17 and CXZ-15 following the manufacturer’s instructions. The protein expression levels of CXCL1, CCL2, CCL5, and CXCL12 in the brain tissues of dogs infected with CGS-17 and CXZ-15 were determined in duplicate using commercial sandwich ELISA kits (Quantikine canine CCL2; CCL5, CXCL1, and CXCL12; R&D Systems, Minneapolis, MN, USA) as previously described [21–24].

### Western blot analysis

Mice were randomly divided into three groups (10 mice per group). Brain tissues were collected from moribund mice infected with the street RABV strains CGS-17 and CXZ-15. The brain samples in each group were lysed and purified with a T-PER Tissue Protein Extraction kit (Pierce). The extracted proteins were separated by SDS-PAGE and then transferred to polyvinylidene difluoride membranes (Millipore Corp). After blocking with 5% nonfat milk in TBST, the membranes were incubated with primary antibodies overnight at 4 °C. The following antibodies were used: anti-p-p38(#4631; Cell Signaling Technology), anti-p38 (#8690; Cell Signaling Technology), anti-p-ERK1/2 (#4370; Cell Signaling Technology), anti-ERK1/2 (#4695; Cell Signaling Technology), anti-p-JNK (#4668; Cell Signaling Technology), anti-JNK (#9258; Cell Signaling Technology), anti-p-NF-κB (#3033; Cell Signaling Technology), anti-NF-κB (#4764; Cell Signaling Technology), anti-IκB (#4812; Cell Signaling Technology), and anti-β-actin (#4970; Cell Signaling Technology). Protein bands were visualized, and the signals were recorded by measuring chemical luminescence (LAS-4000; FUJIFILM).

### Statistical analysis

All of the experiments were performed in at least three independent replicates. Data were analyzed with SPSS 16.0 and GraphPad Prism software (version 6.0). Student’s t-test and one-way analysis of variance (ANOVA) were used to analyze the obtained results, and the results were considered to be statistically significant if *P* < 0.05.

## Results

### Street RABV infection induces chemokine expression in dog and human brain tissues

**Fig. 1:**
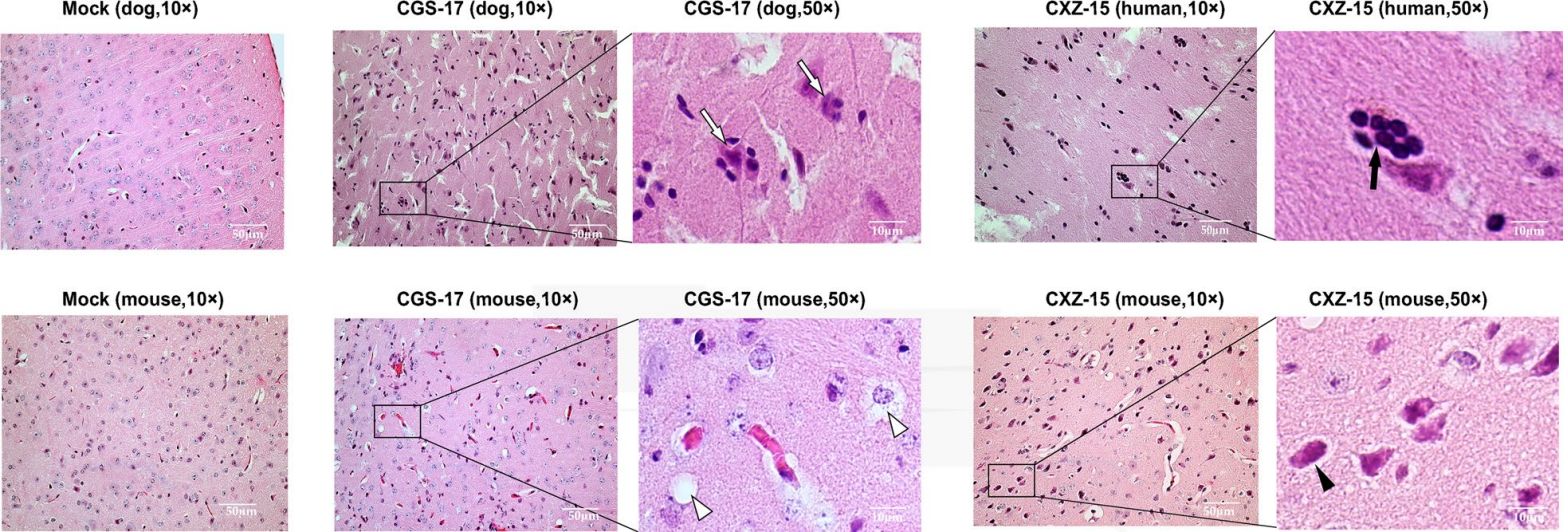
Infection with street RABV strains induces chemokine secretion in mouse brain tissues. **a**–**f** Representative graphs of HE analysis of sections of dog, human, and mouse brains infected with the street RABV strains CGS-17 and CXZ-15. Uninfected dog and mouse brains served as the control group. Scale bars represent 50 μm. ➪ indicates neuronophagy, ➔ indicates formation of glial nodules, ∆ indicates degeneration and necrosis of glial cells, and ▲ indicates neuron degeneration and necrosis

**Fig. 2:**
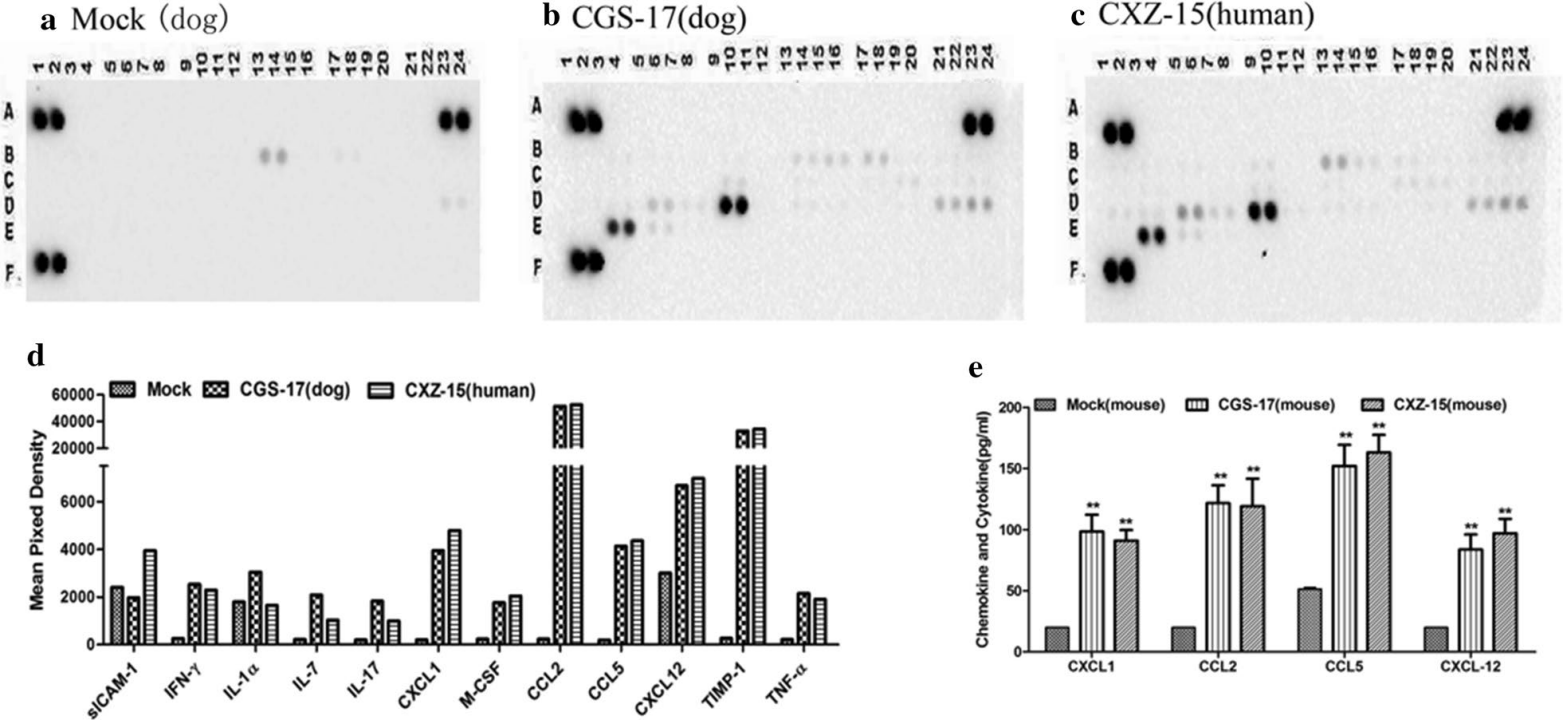
Natural infection with street RABV strains induces chemokine secretion in dog and human brain tissues. **a**–**c** The Proteome Profiler Array Mouse Cytokine Array Panel A was used to detect the protein expression levels of 40 cytokines and chemokines in brain tissues from dogs and humans naturally infected with the street RABV strains CGS-17 and CXZ-15. Uninfected dog brains served as the control group. **d** The quantitative analysis results for the relative signal densities of chemokines in the group of brains infected with CGS-17 and CXZ-15 normalized to those in the control group are shown. **e** The protein expression levels of CXCL1, CCL2, CCL5, and CXCL12 in the brain tissues of mice infected with CGS-17 and CXZ-15 were measured by ELISA. Uninfected mouse brains served as the control group. Data are expressed as the mean ± SD from three independent experiments. *P < 0.05, **P < 0.01, ***P < 0.001, versus the noninfected group

In the present study, we sought to systematically analyze the apparent changes in pathology and inflammatory cytokine expression in dogs and human brain tissues from naturally infected individuals. First, HE staining results showed that increased neuronal degeneration and lymphocyte infiltration were found in the cerebral cortex of dogs and humans that succumbed to rabies. Notably, marked activation of microglial cells, neuronophagy, and formation of glial nodules were observed (Fig. 1a–c). Then, we detected pathological changes in mouse brain tissue infected by the street RABV strains CGS-17 and CXZ-15 and found that congestion of the cortex, degeneration and necrosis of neurons and glial cells, and microglial cell numbers were mildly increased (Fig. 1d–f). To elucidate the inflammatory response induced by street RABV infection in dog and human brain tissues under natural conditions, we next used the ARY006 array to determine the expression levels of cytokines and chemokines. The analysis revealed that the levels of sICAM-1, IFN-γ, IL-1rα, IL-7, IL-17, CXCL1, M-CSF, CCL2, CCL5, CXCL12, TIMP-1 and TNF-α were increased to varying degrees upon CGS-17 and CXZ-15 infection under natural conditions (Fig. 2a–d). As mentioned, we found that the expression of CXCL1, CXCL12, CCL2, CCL5 and TIMP-1 was significantly increased upon CGS-17 and CXZ-15 infection. In addition, the expression of CXCL1, CXCL12, CCL2, and CCL5 in dog brain tissue infected by CGS-17 and CXZ-15 was quantified using canine ELISA kits, as described previously [25], and we found that the levels of CXCL1, CXCL12, CCL5, and CCL2 were obviously increased in street RABV-infected dog brain tissues in relation to healthy tissues (Additional file 2: Figure S2). Then, we detected the expression of CXCL1, CXCL12, CCL2, and CCL5 in mouse brain tissue infected by CGS-17 and CXZ-15 by ELISA, and we found that the expression of these chemokines was upregulated slightly (Fig. 2e). Collectively, our results suggest that natural street RABV infection in dog or human brain tissue induces marked activation of microglia and that degenerated neural cells are surrounded and phagocytosed by microglia, which induces a weak inflammatory response. The degrees of the apparent changes in pathology and inflammatory cytokine expression were obviously increased in the natural infection of dogs or humans compared to artificial model infection of mice.

### Street RABV infection activates the MAPK signaling pathways in dog, human and mouse brain tissues

**Fig. 3:**
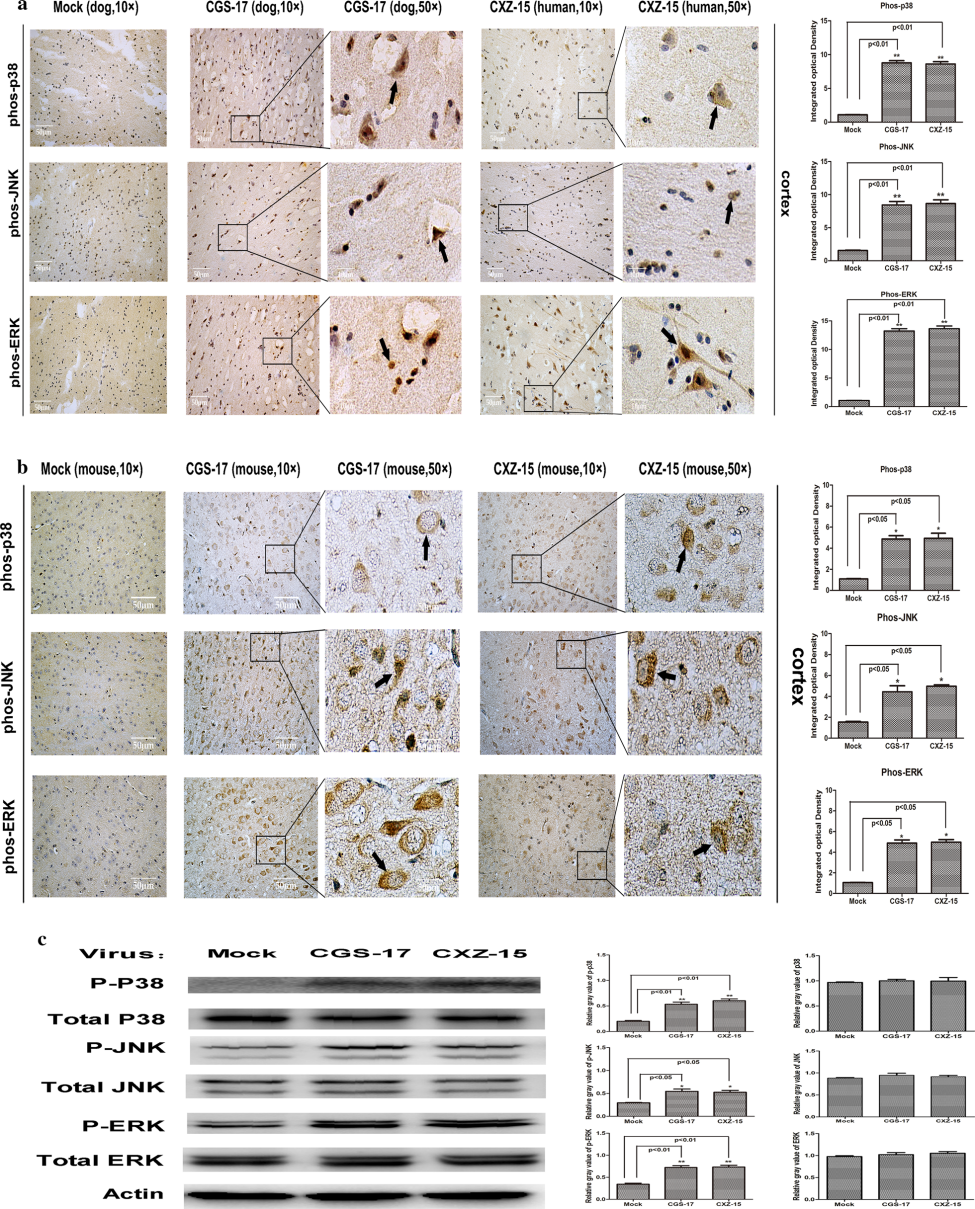
Infection with street RABV strains induces the phosphorylation of p38, JNK, and ERK in dog, human, and mouse brain tissues. **a** Representative graphs of IHC analysis of p-p38, p-JNK, and p-ERK in sections of dog and human brains naturally infected with the street RABV strains CGS-17 and CXZ-15. Uninfected dog brains served as the control group. The quantitative analyses of p-p38, p-JNK, and p-ERK signal densities are shown on the bottom. ➔ indicates positive staining cells. **b** Representative graphs of IHC analysis of p-p38, p-JNK, and p-ERK in sections of mouse brain tissue infected with CGS-17 and CXZ-15. Scale bars represent 50 μm. Uninfected mouse brains served as the control group. The quantitative analysis results for the p-p38, p-JNK, and p-ERK signal densities are shown on the bottom. ➔ indicates positive staining cells. **c** Western blot analysis of mouse brain tissue infected with CGS-17 and CXZ-15. Blots were stained for total p38, total JNK, total ERK, p-p38, p-JNK, p-ERK, and β-actin. The quantitative analysis results for the relative signal densities of total p38, total JNK, total ERK, p-p38, p-JNK, and p-ERK after normalization to the signal density of β-actin are shown on the right. Each test was performed in triplicate. Graphical data represent the mean ± SD. Statistical significance was assessed using one-way ANOVA. *P < 0.05, **P < 0.01, ***P < 0.001

MAPKs play a critical role in the activation of the expression of genes encoding a wide range of cytokines and chemokines in response to viral infection. Thus, we measured the levels of p-p38, p-JNK, and p-ERK1/2 in samples of dog and human brain cortical tissue infected with the street RABV strains CGS-17 and CXZ-15 under natural conditions by IHC assays (Fig. 3a). We found increased p-p38, p-JNK, and p-ERK1/2 signals in dog and human neuronal brain cells upon CGS-17 and CXZ-15 infection. To obtain additional evidence, brain sections from mice infected with CGS-17 and CXZ-15 were examined. Lower levels of MAPK phosphorylation were detected in mouse neuronal brain cells only upon infection with CGS-17 and CXZ-15 (Fig. 3b). We also found only a few immunostained cells undergoing degeneration, and cerebrovascular endothelial cells slightly accumulated in the perivascular spaces of mice infected with street RABV. We next examined the activation of MAPKs in mouse brain tissue infected with CGS-17 and CXZ-15 by WB analysis (Fig. 3c). We found that the expression level of p-JNK was extremely low in the CGS-17 and CXZ-15-infected group; p-p38 and ERK1/2 levels were slightly increased upon infection. Minimal phosphorylation was observed in the control group. These data indicate that the increases in the levels of p-p38, p-JNK, and p-ERK1/2 in infected dog and human brain tissues were induced by street RABV CGS-17 and CXZ-15 and that clear differences in the expression of MAPK pathway components were observed between the dog or human brain in the context of natural infection and the mouse brain in the context of artificial model infection.

### RABV infection activates the NF-κB signaling pathways in dog, human and mouse brain tissues

**Fig. 4:**
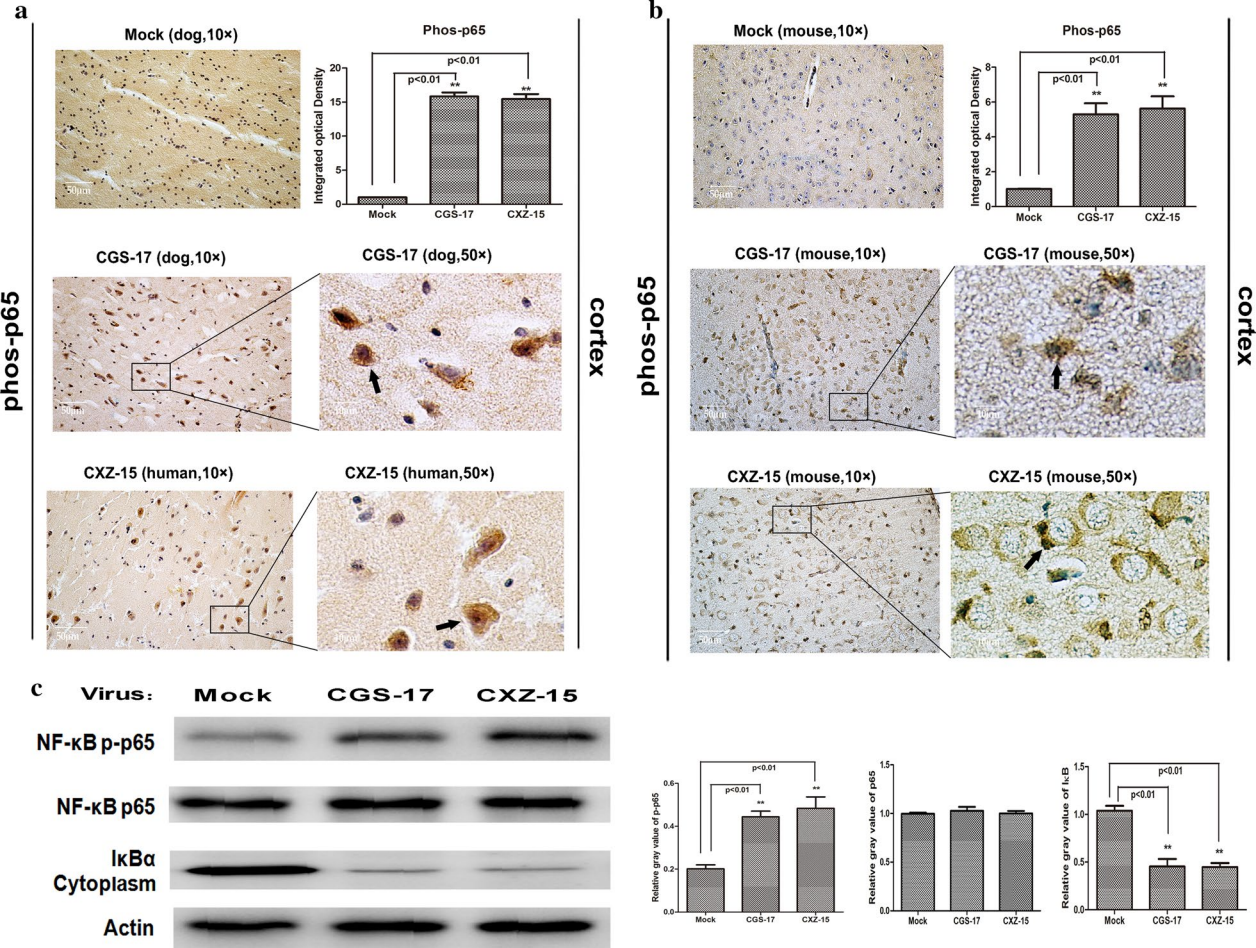
Infection with street RABV strains activates the NF-κB signaling pathway in dog, human, and mouse brain tissues. **a** Representative graphs of IHC analysis of p-p65 in sections of dog and human brains naturally infected with the street RABV strains CGS-17 and CXZ-15. Uninfected dog brains served as the control group. The quantitative analysis results for the p-p65 signal density are shown on the right. ➔ indicates positive staining cells. **b** Representative graphs of IHC analysis of p-p65 in sections of mouse brains infected with CGS-17 and CXZ-15. Uninfected mouse brains served as the control group. Scale bars represent 50 μm. The quantitative analysis results for the p-p65 signal density are shown on the right. ➔ indicates positive staining cells. **c** Western blot analysis of mouse brain tissue infected with CGS-17 and CXZ-15. Blots were stained for p-p65, total p65, IκBα, and β-actin. The quantitative analysis results for the relative signal densities of p-p65, total p65, and IκBα after normalization to the signal density of β-actin are shown on the right. Each test was performed in triplicate. Graphical data represent the mean ± SD. Statistical significance was assessed using one-way ANOVA. *P < 0.05, **P < 0.01, ***P < 0.001

It has been reported that MAPKs contribute to cytokine production by interfering with the nuclear factor kappa B (NF-κB) signaling pathway [26]. Thus, we examined the phosphorylated level of NF-κB p65 in dog and human brain tissue upon natural street RABV CGS-17 and CXZ-15 infection. NF-κB p65 showed a clear distribution in neuronal cells in dog and human brain tissue by IHC assays (Fig. 4a). To assess the activation of the NF-κB signaling pathway in the artificial infection model, mice were infected with street RABV CGS-17 and CXZ-15, and the degree of NF-κB phosphorylation was examined by IHC (Fig. 4b). Increased levels of NF-κB phosphorylation were detected in mouse neuronal brain cells upon infection with the street RABV strains CGS-17 and CXZ-15. Furthermore, the phosphorylation of NF-κB p65 and the degradation of IκBα in mouse brain tissue upon infection with street RABV CGS-17 and CXZ-15 were examined by Western blot analysis (Fig. 4c). We found that NF-κB p65 levels were obviously higher in the brains of street RABV-infected mice than in those of uninfected mice. Additionally, the degradation of IκBα in the cytoplasm was rapidly induced by CGS-17 or CXZ-15 infection. These results show that the phosphorylation of NF-κB p65 following infection with street RABV strains is correlated with the degradation of IκBα in the cytoplasm. Collectively, these results indicate that street RABV strains can increase low NF-κB p65 phosphorylation in the dog, human or mouse brain and that the levels of NF-κB signaling pathway component expression are obviously increased in the context of natural infection in dogs or humans compared with artificial model infection in mice.

## Discussion

RABV is a highly neurotropic virus that usually causes lethal CNS disease, and immune responses and CNS dysfunction are considered to be the main factors during RABV infection [1]. There is little evidence to illuminate the molecular mechanisms of street RABV strains in the context of natural infection. In this study, we showed increased neuronal degeneration, marked activation of microglia, formation of glial nodules and neuronophagy in dog and human brain tissue naturally infected by the street RABV strains CXZ-17 and CXZ-15, but the pathological changes in mouse brain tissue infected by CGS-17 and CXZ-15 were milder. Neurons, microglia and astrocytes are the primary sources of chemokines following infection with a wide range of neurotropic viruses, including RABV, WNV, and herpes simplex virus 1 (HSV-1) [19], and some RABV strains can induce cytokine and chemokine expression in cultured microglia [12]. Several studies have found little tissue damage or histopathological evidence of nervous system dysfunction, and mild inflammation has been observed in a mouse model of street RABV infection; however, T cell and mononuclear cell infiltration into the CNS together with severe encephalitis have been shown in canine experimental models of street RABV infection [3]. Little data exist for rabies studies using street viruses in more representative species, such as dogs or humans. The analysis in this study revealed an increased inflammatory response, and the expression of CXCL1, CXCL12, CCL2 and CCL5 was significantly increased upon natural street RABV strain infection of dogs or humans compared to artificial mouse infection. In the present study, as an initial step toward understanding the status of inflammation, immunopathogenesis related to rabies was compared between dogs or humans and mice. Shuichi et al. reported that the gene structure of canine CXCL12 is similar to that of CXCL12 in humans and other animals, which suggests critical roles in hematopoiesis and leukocyte trafficking in normal canine tissues [24]. In humans and rodents, CCL2 is known to be a potent mediator of the migration of monocytes/macrophages [27]. D.P. Regan et al. demonstrated that the CCL2-CCR2 axis plays an important role in monocyte recruitment in dogs and that overexpression of CCL2 also contributes to other inflammatory changes in the synovial fluid of dogs with canine idiopathic polyarthritis [25, 28, 29]. Both canine and murine bone marrow-derived cultured mast cells (BMCMCs) express mRNA transcripts for CCL5 and MIP-1α and release CCL2 after activation [29]. CXCL1 (also named KC-like) concentrations in the cerebrospinal fluid of dogs with severe inflammation suggest that CXCL1 is specific to inflammation of infectious origin [23, 30]. The association between the brain and CSFAβ_42_ during aging in dogs is similar to that observed in AD patients, and higher Aβ levels in dogs can lead to increased activation of microglia and consequently increased production of CXCL1 [31]. The data obtained here provide direct evidence that the RABV-induced activation of the inflammatory response in the dog infection model more accurately reflects the pathogenic mechanism of human street RABV infection than that in the mouse infection model, which is consistent with the report by Tomoko Ichiki and Karlsson, who demonstrated that the inflammatory and coagulation changes that accompany severe infections in dogs are similar to those observed in humans; large animal models provide very important information, including hemodynamic data and information mimicking human outcomes, that is much closer to human information than that provided by small animal models [23, 32].

The MAPK signaling pathway is activated by diverse groups of viruses [33]. Several studies have demonstrated that laboratory-adapted RABV-induced expression of CXCL10 and CCL5 is achieved by activation of the p38 and NF-κB pathways [12]. Our study showed that the increased phosphorylation of p38, JNK and ERK1/2 was detected in neuronal cells only upon street RABV infection in dog and human brain tissues, but lower expression of MAPK signaling pathway components was observed in the mouse brain in the context of street RABV infection. A previous study showed that preserving the integrity of infected neurons by limiting viral replication and subsequently reducing glycoprotein expression in the CNS contributed to street RABV immune evasion and limited proinflammatory cytokine or chemokine production in a mouse model [34], which may be the reason for mild inflammation and limited signal transduction in this model. Several studies have also reported that the enhanced signaling activity may be appropriated by viruses to support effective replication. p38 phosphorylation has been found to regulate the expression of CCL5 in HIV infection and is associated with brain injury, while activation of JNK induces the secretion of proinflammatory cytokines and is exploited for viral replication [11, 35, 36]. Thus, we suspect that the induction of the inflammatory response and MAPK activation is related to brain injury in dogs and humans in the context of natural street RABV infection.

Viruses have evolved many strategies to manipulate the NF-κB pathway for their own benefit. Activation of p38 and JNK was reported to regulate the inflammatory response to viral infections mediated by NF-κB, c-Jun, and STAT1 [26]. Our study found that the phosphorylation of NF-κBp65 was obviously distributed in neuronal cells in dog and human brain tissues naturally infected by street RABV compared to those in brain tissues in a mouse infection model. Previous data demonstrated that the expression of CXCL10 and CCL5 in RABV-infected microglia was strongly mediated by MAPK and NF-κB and that NF-κB signaling upon RABV infection was augmented via a p38-mediated mechanism. IFNs secreted from RABV-infected microglia stimulate IFN signaling in an autocrine manner, which subsequently facilitates chemokine gene expression in cooperation with NF-κB [12]. Interestingly, our studies were largely consistent with the status of the MAPK pathway, and some studies have also demonstrated that the RABV-induced activation of MAPK may activate one or more of the signaling molecules upstream of IκB in the NF-κB cascade, which subsequently induces NF-κB activation in response to proinflammatory cytokines [26]. We found that the level of IκB in the cytoplasm was reduced, resulting in NF-κB activation upon street RABV infection. Thus, we predict that the NF-κB pathway in brain tissues is indirectly augmented upon street RABV infection via the activation of the MAPK pathway, which is similar to a previous study on RABV and CHPV [12]. Considering the strong effects of the significantly increased levels of CXCL1, CXCL12, CCL2 and CCL5 induced by street RABV infection on a broad range of inflammatory cells, the induction of chemokines and their roles in orchestrating downstream immune events are a key feature in clearing RABV from the CNS [18]. The MAPK and NF-κB signaling pathways intrinsically adjust the level of chemokine expression via recognition of viral infection, thereby controlling excessive leukocyte trafficking into the brain. In addition, street RABV induces recruitment of relatively few inflammatory cells into the CNS by limiting the expression of cytokines and chemokines and maintaining blood–brain barrier integrity [37]. Our research again provides evidence for the association of street RABV infection with the MAPK and canonical NF-κB pathways in dogs, humans and mice. It would therefore be fundamental to further investigate the associations of street RABV infection with blood–brain barrier integrity and the MAPK/NF-κB pathways in dogs, humans or mice.

There are known biological differences among strains of rabies that can influence the host’s ability to initiate innate immune responses, but in most cases, the molecular basis of these differences is not clear [13]. The mouse model of rabies infection is a convenient and economical research model for studying the pathogenesis of rabies and the development of vaccines or diagnostic reagents. However, the pathological changes observed in experimental infection models greatly differ among the various viral strains [15], and the abnormalities caused by laboratory-adapted RABV strain infection are not present in all infected animals, indicating that these strains do not reflect the pathogenetic process and mechanism in dogs or humans infected with street rabies virus under natural conditions [14]. In developing studies to explain the pathogenesis of rabies more accurately, it is important to note that the dog model more closely mimics the real-world situation of infection with a street strain of rabies.

Overall, our data demonstrate that street RABV infection can stimulate inflammatory responses and MAPK and NF-κB activation in the context of natural infection in dog and human brain tissues and the degree of inflammation is more obvious in dog and human tissues than in the corresponding tissues in the mouse infection model tested. These observations have led to renewed efforts to obtain evidence of the mechanisms underlying fatal natural RABV infection.

## Conclusions

The apparent changes in the pathology of RABV infection and associated immune responses are obviously higher in natural dog or human infections than in artificial mouse models. Comparative studies indicate that compared with a mouse model, natural street RABV infection in dog or human brain tissue induces increased neuronal degeneration, cell infiltrates, marked microglial activation and neuronophagy, which significantly increases the expression of CXCL1, CXCL12, CCL2 and CCL5. Chemokines and cytokines produced in RABV infection are crucial for RABV clearance from the CNS through the modulation of leukocyte recruitment into the CNS and activation of various immune cells and signaling pathways that are essential for viral clearance and protection. In RABV infection, increases in the levels of p-p38, p-JNK, and p-ERK1/2 occur in infected dog and human brain tissues, but lower levels of MAPK phosphorylation are observed only in mouse neuronal brain cells upon infection, and only a few immunostained degenerating cells and cerebrovascular endothelial cells accumulate to low numbers in the perivascular spaces of mice infected with street RABV. Moreover, the phosphorylation of NF-κB p65 shows a clear distribution in neuronal cells in dog and human brain tissue, following infection with street RABV strains, which is correlated with the degradation of IκBα in the cytoplasm. The level of the expression of NF-κB signaling pathway components is obviously increased in natural infected dogs and humans compared with artificially infected model mice. The data obtained here provide direct evidence for the RABV-induced activation of the inflammatory response in a dog infection model and indicate that the dog model more accurately reflects the pathogenic mechanism of human street RABV infection than does the mouse model. The present study provides insight into the precise roles of mechanisms underlying fatal natural RABV infection.

## Supplementary information

**Additional file 1: Figure S1.** Mouse Cytokine Array coordinates. This image is for coordinate reference, using the transparency overlay for analyte identification. Please refer to the table for the mouse cytokine array coordinates.

**Additional file 2: Figure S2.** Natural infection with street RABV strains induces CXCL1, CCL2, CCL5, and CXCL12 secretion in dog brain tissues. The protein expression levels of CXCL1, CCL2, CCL5, and CXCL12 in the brain tissues of dogs infected with CGS-17 and CXZ-15 were measured by ELISA. Uninfected dog brains served as the control group. Data are expressed as the mean ± SD from three independent experiments. *P < 0.05, **P < 0.01, ***P < 0.001 versus the noninfected group.

## Data Availability

The materials described in the manuscript will be made freely available to any scientist wishing to use them.

## Abbreviations

RABV: Rabies virus
CNS: Central nervous system
MAPK: Mitogen-activated protein kinase
NF-κB: Nuclear factor kappa B
p38: P38 kinase
JNK: C-Jun-N-terminal kinase
ERK1/2: Extracellular-regulated kinase
HE: Hematoxylin and eosin
IHC: Immunohistochemistry
