# Supplementary figures and images for: Inflammatory response and MAPK and NF-κB pathway activation induced by natural street rabies virus infection in the brain tissues of dogs and humans
Source: Virol J. 2020 Oct 20;17:157. doi: 10.1186/s12985-020-01429-4 (PMC7576862; doi:10.1186/s12985-020-01429-4)

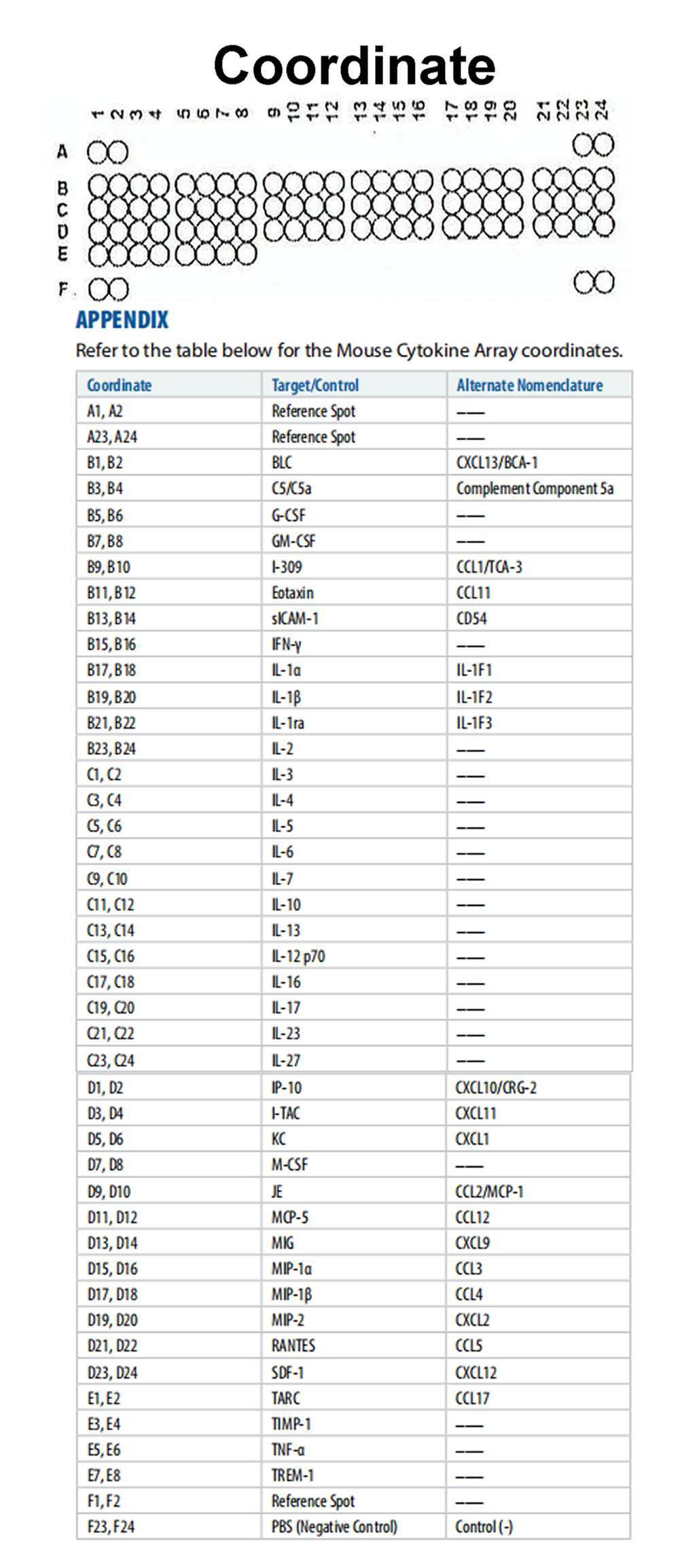

Supplement: Supplementary file 1 — Additional file 1: Figure S1. Mouse Cytokine Array coordinates. This image is for coordinate reference, using the transparency overlay for analyte identification. Please refer to the table for the mouse cytokine array coordinates. [file 12985_2020_1429_MOESM1_ESM.tif]

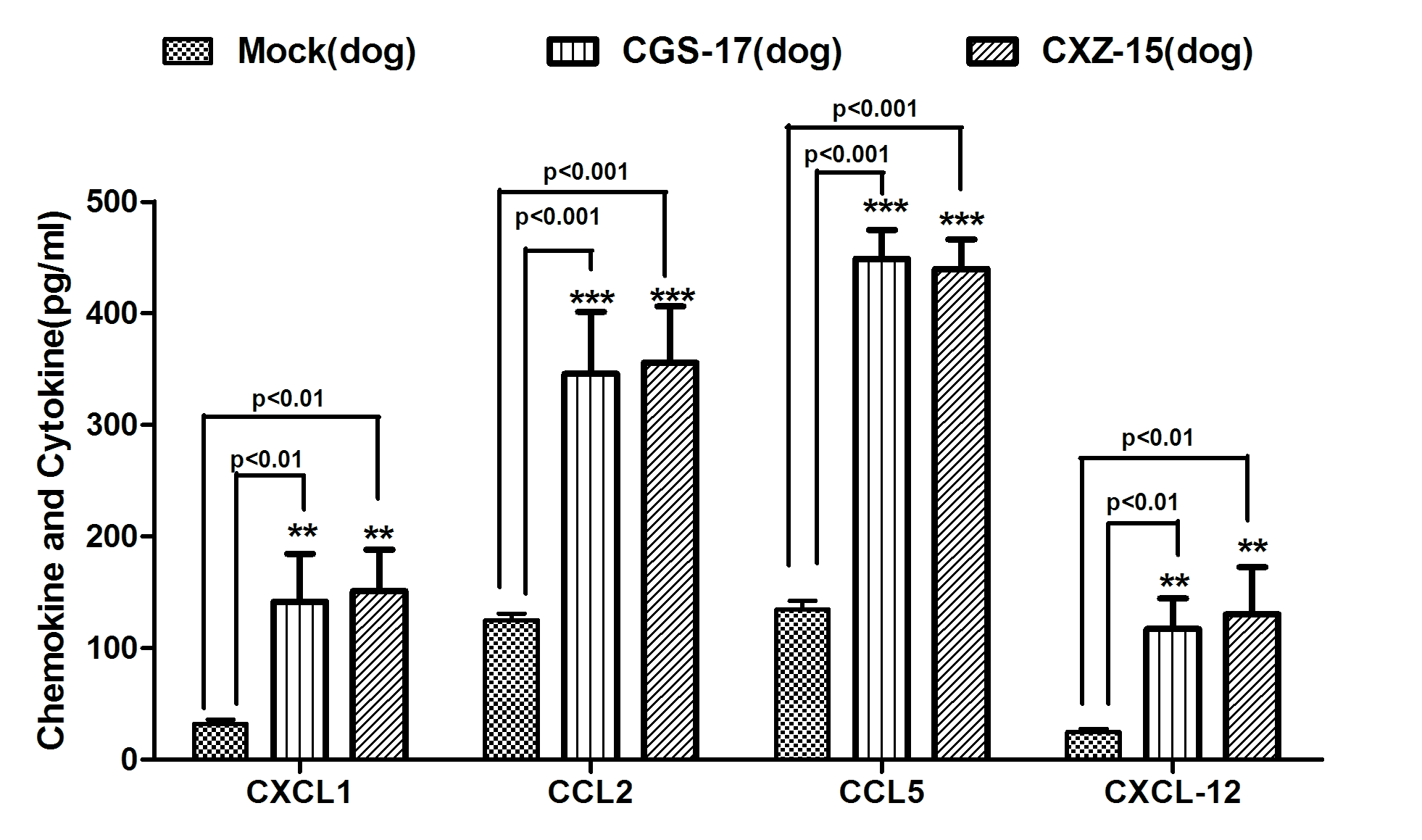

Supplement: Supplementary file 2 — Additional file 2: Figure S2. Natural infection with street RABV strains induces CXCL1, CCL2, CCL5, and CXCL12 secretion in dog brain tissues. The protein expression levels of CXCL1, CCL2, CCL5, and CXCL12 in the brain tissues of dogs infected with CGS-17 and CXZ-15 were measured by ELISA. Uninfected dog brains served as the control group. Data are expressed as the mean ± SD from three independent experiments. *P < 0.05, **P < 0.01, ***P < 0.001 versus the noninfected group. [file 12985_2020_1429_MOESM2_ESM.tif]
